# Gut bacteria differentially affect egg production in the anautogenous mosquito *Aedes aegypti* and facultatively autogenous mosquito *Aedes atropalpus* (Diptera: Culicidae)

**DOI:** 10.1186/s13071-016-1660-9

**Published:** 2016-06-30

**Authors:** Kerri L. Coon, Mark R. Brown, Michael R. Strand

**Affiliations:** Department of Entomology, The University of Georgia, 120 Cedar Street, 420 Biological Sciences, Athens, GA 30602 USA

**Keywords:** Microbiota, Development, Reproduction, Oogenesis, Clutch size

## Abstract

**Background:**

*Aedes aegypti* and *A. atropalpus* are related mosquitoes that differ reproductively. *Aedes aegypti* must blood-feed to produce eggs (anautogenous) while *A. atropalpus* always produces a first clutch of eggs without blood-feeding (facultatively autogenous). We recently characterized the gut microbiota of *A. aegypti* and *A. atropalpus* that were reared identically in the laboratory. Here, we assessed the effects of specific members of the gut microbiota in *A. aegypti* and *A. atropalpus* on female fitness including egg production.

**Methods:**

Gnotobiotic *A. aegypti* and *A. atropalpus* larvae were colonized by specific members of the gut microbiota. Survival, development time, size and egg production for each treatment was then compared to axenic and conventionally reared larvae.

**Results:**

Most species of bacteria we tested supported normal development and egg production by *A. aegypti* but only one betaproteobacterium, a *Comamonas*, supported development and egg production by *A. atropalpus* to equivalent levels as conventionally reared females. *Aedes atropalpus* females colonized by *Comamonas* contained similar stores of glycogen and protein as conventionally reared females, whereas females colonized by *Aquitalea* did not. Small differences in bacterial loads were detected between gnotobiotic and conventionally reared *A. aegypti* and *A. atropalpus*, but this variation did not correlate with the beneficial effects of *Comamonas* in *A. atropalpus*.

**Conclusions:**

Specific members of the gut microbiota more strongly affected survival, size and egg production by *A. atropalpus* than *A. aegypti*.

**Electronic supplementary material:**

The online version of this article (doi:10.1186/s13071-016-1660-9) contains supplementary material, which is available to authorized users.

## Background

Most mosquito species are anautogenous, which means that after mating with males, adult females must consume at least one blood meal from a vertebrate host for each clutch of eggs they produce and lay [1]. Repeated cycles of blood-feeding and egg production also underlie why several mosquito species have evolved into vectors that transmit pathogens that cause several serious diseases in humans and other animals. In contrast, some mosquito species are autogenous and produce eggs without blood-feeding [1]. Facultatively autogenous species emerge as adults and lay a first clutch of eggs without blood-feeding but must blood-feed to produce additional clutches. Obligately autogenous species never blood-feed.

Regulation of egg formation is best understood in the anautogenous mosquito *Aedes aegypti*, which vectors several human pathogens including the viruses that cause yellow fever, dengue fever and Zika virus disease*.* In brief, the fat body of newly emerged *A. aegypti* females becomes competent to produce yolk proteins through the activity of juvenile hormone [2, 3]. However, oogenesis remains arrested until a blood meal is taken, which triggers the release of two neurohormones from the brain, insulin-like peptides (ILPs) and ovary ecdysteroidogenic hormone (OEH), that stimulate the ovaries to produce ecdysone [4, 5]. Ecdysone, ILPs and other factors regulate the fat body to produce yolk proteins that are packaged into primary oocytes to produce mature eggs [4–8]. Nutrients in the blood meal together with teneral reserves acquired during larval feeding provide the resources needed to produce yolk proteins [9–11].

The rockpool mosquito, *Aedes atropalpus*, is closely related to *A. aegypti* [12] but is facultatively autogenous. *Aedes atropalpus* females produce similar numbers of eggs in the first gonadotropic cycle as *A. aegypti*, but fully rely on nutrient reserves acquired from feeding during the larval stage to do so [10, 13–15]. ILPs and OEH play a similar role in stimulating the ovaries in *A. atropalpus* to produce ecdysone and the fat body to synthesize yolk proteins as determined for *A. aegypti* [15, 16]. What differs is that *A. atropalpus* females release these neurohormones shortly after emerging as adults rather than in response to blood-feeding [15, 16]. The mechanism(s) that triggers blood meal-independent release of these hormones remains unclear, although data suggest it is linked to nutrient sensing and the availability of sufficient teneral reserves to support egg production [10, 15, 16].

Microorganisms in the digestive tract can also influence nutrient acquisition by animals including insects [17]. Mosquitoes host low diversity bacterial communities in their digestive tract that consist primarily of gram-negative aerobes and facultative anaerobes [18–25]. Mosquitoes acquire most if not all of these bacteria from the aquatic habitat they develop in as larvae while transmitting some but not all community members to adults [19, 21].

We recently used 16S rDNA sequencing to characterize the gut microbiota of *A. aegypti* and *A. atropalpus* larvae reared under identical environmental conditions in the laboratory. Results showed that similar communities of bacteria were present in the aquatic habitat of both species during larval development. However, Bacteroidetes (Flavobacteriaceae) and Actinobacteria (Microbacteriaceae) dominated the gut community in *A. aegypti* larvae, whereas Proteobacteria (Class Betaproteobacteria) dominated the community in *A. atropalpus* [21]. We developed methods to produce axenic (i.e. bacteria-free) larvae, which showed that both species fail to develop beyond the first instar in the absence of gut bacteria, but develop normally into adults if recolonized by bacteria in their laboratory aquatic habitat. We also developed methods to produce gnotobiotic larvae that were colonized by a particular species of bacterium. Bioassays showed that several species of bacteria present in the laboratory aquatic habitat could individually colonize axenic *A. aegypti* larvae, and that the resulting gnotobiotic larvae also develop normally into adults [21]. However, no studies have examined the effects of the gut microbiota in larvae on egg production by *A. aegypti* or any fitness traits in *A. atropalpus*.

In this study, we produced gnotobiotic *A. aegypti* and *A. atropalpus* larvae that were singly colonized by abundant members of the larval gut microbiota and compared their effects on female-related fitness traits including egg production in the first ovarian cycle. Our results showed that gnotobiotic *A. aegypti* singly colonized by several members of the gut microbiota developed and produced eggs similarly to conventionally reared *A. aegypti* females with a mixed community of bacteria. In contrast, only one community member we tested rescued development and egg production to equivalent levels as conventionally reared *A. atropalpus* females*.*

## Methods

### Conventional rearing of *A. aegypti* and *A. atropalpus*

The UGAL strain of *A. aegypti* and Bass Rock strain of *A. atropalpus* were conventionally reared in the same insectary at 27 °C, 60 % relative humidity, and 16 h light: 8 h dark photoperiod [16]. Larvae were fed a standard diet consisting of finely ground rat chow (Purina): lactalbumin: brewers yeast (1:1:1) in open aluminum rearing pans containing distilled water [26]. Pupae were transferred from larval rearing pans to plastic cages for adult emergence. These methods produced large cohorts of adult *A. aegypti* and *A. atropalpus* that were similar in size. Adults were fed 10 % sucrose in water (wt/vol) *ad libitum*. Adult female *A. aegypti* were blood-fed two days post-emergence on an anesthetized rat until engorged. *Aedes aegypti* females laid a clutch of eggs approximately 36 h after blood-feeding on damp filter paper. *Aedes atropalpus* females in contrast laid clutches of eggs on damp filter paper 3–5 days post-emergence.

### Isolation and taxonomic assignment of bacteria from larvae

Bacterial isolates were maintained as summarized in Additional file 1: Table S1. Bacteria were isolated from conventionally reared *A. aegypti* and *A. atropalpus* larvae by collecting and surface sterilizing fourth instars followed by homogenization in 1.6 ml centrifuge tubes in sterile phosphate buffered saline (PBS) using a plastic pestle [21]. Homogenates were then plated on Luria broth (LB), brain-heart infusion (BHI), tryptic soy agar (TSA), Reasoner’s 2A (R2A), or blood agar plates at 27 °C for 24–72 h (Additional file 1: Table S1). Single colonies were picked for serial dilution streaking followed by DNA isolation from a single colony using the Gentra Puregene Yeast/Bact kit (Qiagen). After PCR amplification using universal primers (1492F and 129R in Additional file 1: Table S2) and cloning into a vector (pCR 2.1 TOPO TA cloning, Invitrogen), ~1,000 bp of the 16S–23S internal transcribed spacer (ITS) region was Sanger sequenced. Resulting sequence data were then used to design genus-specific primers for each isolate of interest (Additional file 1: Table S2). Specificity of primers for a given isolate was verified by PCR using DNA from each of the identified bacterial isolates as previously described [21].

### Inoculation of axenic first instars with particular gut community members

Axenic larvae were produced by surface sterilizing eggs from the conventionally reared *A. aegypti* and *A. atropalpus* colonies followed by hatching of first instars in sterile water per Coon et al. [21]. Larval diet was sterilized by exposure to 5 kGy from a cobalt 60 gamma radiation source, while 10 % sucrose was filtered sterilized for feeding adults. First instars were inoculated with a particular species of bacterium by placing 20 axenic larvae in a 150 × 15 mm Petri dish containing water sterilized by autoclaving followed by addition of sterile diet and approximately 10^8^ cells of a given bacterial isolate suspended in sterile water. Sterilized diet was thereafter added to dishes every other day until death or pupation. Pupae were surface-sterilized by placing in 2 % (vol/vol) bleach for 2 min and rinsing 3 times in sterile water. Pupae were then placed in sterile water in an autoclaved polypropylene plastic chamber (Olcott Plastics) for adult emergence. The axenic status of larvae and food were confirmed by culture-based methods and PCR analysis using universal 16S rRNA primers (27F and 1492R in Additional file 1: Table S2) [21]. The same methods were also used to determine the presence of bacteria or a particular isolate in water, larvae or adults.

### Larval survival and development time assays

Dishes containing 20 first instars inoculated with a particular bacterial isolate were produced as described above. Dishes containing 20 axenic first instars with sterile food but no bacteria served as a negative control, while 20 conventional first instars in non-sterile water but fed sterile food served as the positive control. Dishes were then maintained under the same environmental conditions. The number of larvae that pupated per dish for each treatment was recorded daily. All pupae from a dish were then removed, surface sterilized, and placed in sterile water in sterile containers as described above. Dead larvae and pupae were removed and discarded. Survival to adulthood was measured as the proportion of first instars that successfully emerged as adults. Between 5 and 30 replicate dishes were monitored for each treatment. With 20 larvae per replicate, we overall monitored from 100 to 600 first instars per treatment.

### Adult size and mature egg production assays

Gnotobiotic and conventionally reared adult females were transferred to polypropylene cages (Olcott Plastics) lined with moist filter paper and containing a cotton wick soaked with water. Filter paper, wicks, water and cages were autoclaved before use. The number of eggs laid by each female was counted by visually inspecting the filter paper using a dissecting microscope. Each female was then dissected in PBS and the number of mature eggs remaining in the ovaries was also determined by visual inspection using previously established criteria [15]. These criteria included that unlaid mature eggs had to be ≥ 300 μm in length and have a fully formed chorion. Egg production was quantified for individual, mated *A. atropalpus* females at 120 h post-emergence. Egg production was quantified for individual, mated *A. aegypti* females 72 h after blood-feeding on a surface-sterilized rat. First clutch size of each female was defined as the total number of oviposited plus mature eggs remaining in the ovaries. The size of each ovipositing female was then determined by measuring the length of the forewing from the axillary incision to the tip excluding fringe using an ocular micrometer. A minimum of 20 females per treatment was assayed.

### Nutrient reserves in conventional and gnotobiotic *A. atropalpus*

Conventionally reared and gnotobiotic adult females were collected 12 h post-emergence followed by dissection in PBS and removal of the digestive tract. The remaining body wall, which contains the fat body, from two individuals was transferred to a microfuge tube and homogenized in 100 μl of water (protein assay) or 100 μl of Na_2_SO_4_ with 200 μl of methanol (glycogen, lipid assays) followed by storage at −80 °C before use [27]. After centrifugation (12,000× *g*, 4 °C), supernatants were used to determine total protein, glycogen and lipid amounts as previously described [4, 16]. A total of 10 body wall pairs were assayed for each treatment.

### Bacterial load in conventional and gnotobiotic *A. aegypti* and *A. atropalpus*

The number of bacteria in gnotobiotic larvae and adults was assessed by standard plate counts. Individual larvae were collected after molting to the fourth instar from replicate dishes, surface-sterilized, homogenized in 100 μl of PBS as described above, serially diluted and plated on LB agar. Colony forming units (CFUs) were then determined after incubation of plates at 27 °C for 24 h. Homogenates were also plated from adult females collected 6–12 h and either 24 h post-blood meal (*A. aegypti*) or 72 h post-emergence (*A. atropalpus*). At least 4 larvae or adults were analyzed per treatment with each sample internally replicated 4 times.

### Statistical analysis

All analyses were performed using R (http://www.r-project.org/). Development time and total nutrient amounts were tested for normality and equality of variances before analysis by one-way analysis of variance (ANOVA), followed by *post-hoc* comparisons using Dunnett’s tests. Survival data were analyzed by Chi-square or Fisher’s exact tests followed by *post-hoc* Bonferroni-corrected pairwise tests to compare treatments. Bacterial counts were analyzed by ANOVA followed by either *post-hoc* comparison to the conventional positive control using Dunnett’s test or Tukey-Kramer Honest Significant Difference (HSD) tests. The number of mature eggs females produced was analyzed by Fisher’s exact test to compare the proportion of females that produced one or more mature eggs in each bacterial treatment while the effects of size were analyzed by logistic regression. These data were also examined by an analysis of covariance (ANCOVA) followed by *post-hoc* comparisons using a Dunnett’s test.

## Results

### Individual bacteria differentially affect *A. aegypti* and *A. atropalpus* development

We first compared development time to pupation and survival to adulthood of gnotobiotic larvae colonized by individual bacterial isolates to that of conventionally reared control larvae. We focused our assays on representative genera from each of the major bacterial phyla previously identified from the digestive tracts of our laboratory mosquitoes ([21]; Additional file 1: Table S1): *Paenibacillus* (Firmicutes: Paenibacillaceae), *Chryseobacterium* (Bacteroidetes: Flavobacteriaceae), *Sphingobacterium* (Bacteroidetes: Sphingobacteriaceae), *Microbacterium* (Actinobacteria: Microbacteriaceae), *Leucobacter* (Actinobacteria: Microbacteriaceae), *Aquitalea* (Proteobacteria: Neisseriaceae) and *Comamonas* (Proteobacteria: Comamonadaceae)*.* Two of these genera, *Aquitalea* and *Comamonas*, belong to the Betaproteobacteria and were previously identified as dominant members of the *A. atropalpus* larval gut community by pyrosequencing. All other genera were previously isolated from *A. aegypti* ([21]; Additional file 1: Table S1). Members of the Bacteroidetes (Flavobacteriaceae, Sphingobacteriaceae) and Actinobacteria (Microbacteriaceae) are commonly found in anautogenous species both in the lab and field [18–25], and make up as much as 90 % of the gut bacterial community in our laboratory mosquito cultures [21].

We collected eggs from conventionally reared *A. aegypti* and *A. atropalpus* and produced axenic (i.e. bacteria-free) first instars, which were either maintained in sterile water with sterile food or placed into water inoculated with one of the above isolates plus food. PCR screening of bacterial 16S rDNA using universal primers 6 h after hatching confirmed the presence of bacteria in conventionally reared first instars and the absence of bacteria in axenic larvae (Additional file 1: Figure S1). Screening with genus specific primers also confirmed that inoculation of axenic larvae with the above isolates resulted in larvae that contained each bacterium at 6 h post-inoculation (Additional file 1: Figure S1).

**Fig. 1:**
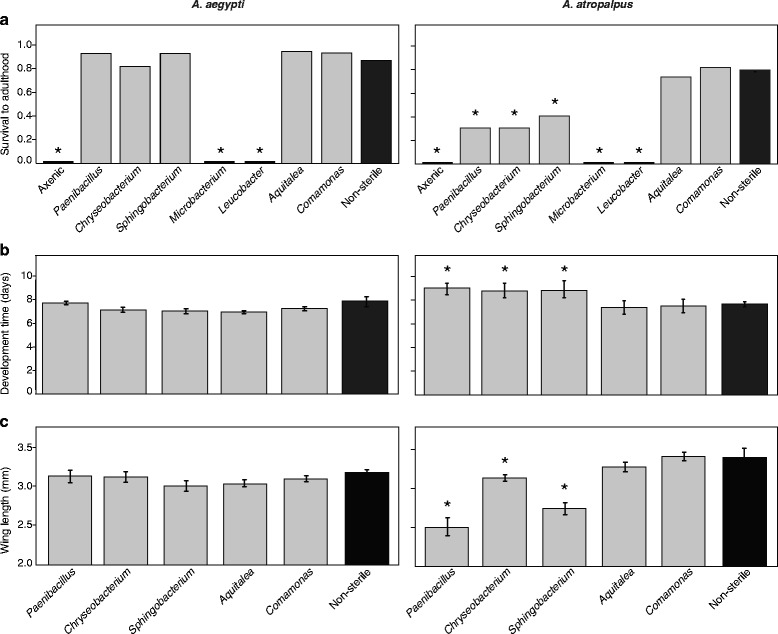
Development of *Aedes aegypti* and *A. atropalpus* larvae that were axenic, inoculated with a single bacterial species, or conventionally reared (non-sterile). **a** Survival from egg hatching to adult emergence differed among treatments for *A. aegypti* (Fisher’s exact test: *P* = 0.0005) and *A. atropalpus* (Fisher’s exact test: *P* < 0.0001). An asterisk (*) above a given bar indicates the treatment significantly differed from the non-sterile control by post-hoc pair-wise comparisons with Bonferroni correction. **b** Development time from egg hatching to pupation differed among treatments for *A. aegypti* (ANOVA: *F*_(5,673)_ = 8.0, *P* < 0.0001) and *A. atropalpus* (ANOVA: *F*_(5,880)_ = 211.3, *P* < 0.0001). **c** Size as estimated by forewing length did not differ among treatments for *A. aegypti* (ANOVA: *F*_(5,183)_ = 1.2, *P* = 0.29) but did differ for *A. atropalpus* (ANOVA: *F*_(5,129)_ = 32.7, *P* < 0.0001). Asterisks above the bars in (**b**) or (**c**) indicate means that significantly differ from the non-sterile control as determined by Dunnett’s test (*P* < 0.01). A minimum of 5 replicate dishes and 100 larvae per treatment were assayed for survival and development times. A single forewing from a minimum of 20 randomly selected adult females per treatment was measured to estimate adult size. The bars in (**b**) and (**c**) present mean values with 95 % confidence intervals for each treatment

Results showed that 87 % of *A. aegypti* and 82 % of *A. atropalpus* larvae reared conventionally (= non-sterile) developed and emerged as adults at 8 days post-egg hatching (positive control), while as previously reported no axenic *A. aegypti* or *A. atropalpus* larvae developed beyond the first instar (negative control) (Fig. 1a). All axenic *A. aegypti* and *A. atropalpus* that were inoculated with *Microbacterium* or *Leucobacter* also died 4–5 days post-inoculation as first instars (Fig. 1a). All of the other isolates tested supported survival to adulthood and development times for *A. aegypti* larvae that did not significantly differ from conventionally reared larvae (Fig. 1a, b). In contrast, only *Aquitalea* and *Comamonas* supported survival and development times for *A. atropalpus* larvae that did not differ from conventionally reared *A. atropalpus* (Fig. 1a, b). Survival of *A. atropalpus* larvae inoculated with *Paenibacillus*, *Chryseobacterium* or *Sphingobacterium* was intermediate between the negative and positive controls with development times for survivors also being longer than for conventionally reared *A. atropalpus* (Fig. 1a, b). The reduced survival of *A. atropalpus* inoculated with *Paenibacillus*, *Chryseobacterium* or *Sphingobacterium* was due to a larger proportion of individuals dying as larvae (*χ*^2^ = 18.9054, *df* = 1, *P* < 0.0001) rather than a smaller proportion of pupae failing to emerge as adults (*χ*^2^ = 0.1822, *df* = 1, *P* > 0.05).

For the individuals in each treatment that developed into adults, we estimated female size by measuring forewing length, which has been used in several other studies of mosquitoes including *A. aegypti* and *A. atropalpus* to estimate body size [10, 28, 29]. There were no differences in size between conventionally reared *A. aegypti* adult females and adults that emerged from larvae colonized by *Paenibacillus*, *Chryseobacterium*, *Sphingobacterium*, *Aquitalea* or *Comamonas* (Fig. 1c). However, only gnotobiotic *A. atropalpus* larvae inoculated with *Aquitalea* or *Comamonas* developed into adults that did not significantly differ in size from conventionally reared *A. atropalpus* (Fig. 1c). *Aedes atropalpus* adults produced from larvae inoculated with *Paenibacillus*, *Chryseobacterium* or *Sphingobacterium* were significantly smaller (Fig. 1c).

### Several bacteria support normal egg production in *A. aegypti* but not *A. atropalpus*

We next focused on those species of bacteria that supported development of at least some *A. aegypti* and *A. atropalpus* to adulthood by assessing their effects on egg production by females. For each treatment, females were mated with males from the same cohort and subsequently housed individually in sterile containers for 36–120 h. *Aedes aegypti* females were blood-fed 2 days post emergence. Egg production data were recorded in three ways: (i) the proportion of females for each treatment that laid or contained at least one mature egg; (ii) the total number of mature eggs per female (laid plus present in the ovaries); and (iii) the total number of laid eggs.

**Fig. 2:**
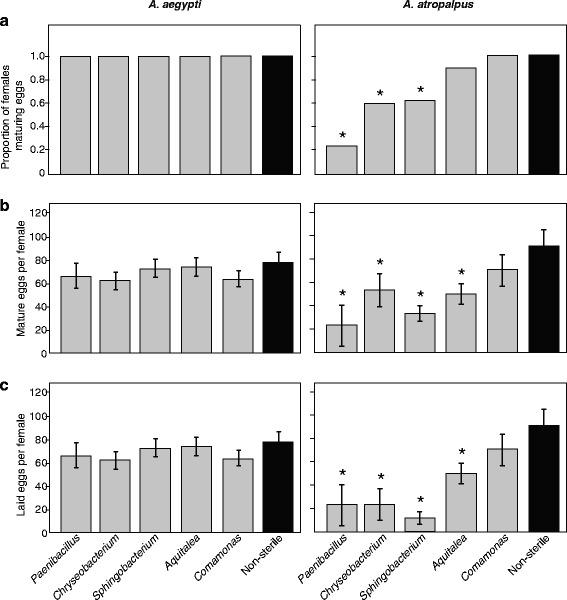
Mature egg formation by *Aedes aegypti* and *A. atropalpus* adult females from larvae that were inoculated with a single bacterial species or conventionally reared (Non-sterile). **a** The proportion of females that produced one or more mature eggs did not differ among treatments for *A. aegypti* (Fisher’s exact test: *P* > 0.05) but did differ for *A. atropalpus* (Fisher’s exact test: *P* < 0.0001). An asterisk above a given bar (*) indicates the treatment significantly differed from the non-sterile control by post-hoc pair-wise comparisons with Bonferroni correction. **b** Total clutch sizes (sum of the number of eggs laid and the number of mature eggs in the ovaries) did not differ among treatments for *A. aegypti* (ANOVA: *F*_(5,183)_ = 2.3, *P* > 0.05) but did differ for *A. atropalpus* (ANOVA: *F*_(5,127)_ = 13.0, *P* < 0.0001). **c** Number of eggs laid by females in a given treatment that produced at least one mature egg did not not differ for *A. aegypti* (ANOVA: *F*_(5,183)_ = 2.2, *P* > 0.05) but did differ for *A. atropalpus* (ANOVA: *F*_(5,127)_ = 18.4, *P* < 0.0001). Bars in (**b**) and (**c**) present mean values with 95 % confidence intervals while asterisks (*) in (**b**) and (**c**) indicate treatments that significantly differ from the non-sterile control (Dunnett’s test; *P* < 0.01)

For *A. aegypti*, all females in each treatment produced mature eggs (Fig. 2a). All gnotobiotic females also produced the same total number of mature eggs (*F*_(5,183)_ = 2.26, *P* > 0.05) and laid the same number of eggs (*F*_(5,183)_ = 2.19, *P* > 0.05) as conventionally reared females (Fig. 2b, c). In contrast, there was a very strong treatment effect on the proportion of *A. atropalpus* females that produced any mature eggs (Fisher’s exact test; *P* < 0.0001) (Fig. 2a). Almost all conventionally reared females and females colonized by *Comamonas* or *Aquitalea* produced mature eggs, whereas < 60 % of gnotobiotic females colonized by *Chryseobacterium* or *Sphingobacterium* produced eggs while only 23 % of females colonized by *Paenibacillus* produced eggs (Fig. 2a). We considered that these differences could reflect the effect of adult size rather than bacterium. However, regression analysis across all individuals showed that wing length did not strongly predict whether a female produced mature eggs (*P* = 0.08). For the *A. atropalpus* females in each treatment that produced mature eggs, we compared clutch sizes using an ANCOVA where wing length served as the covariate. We detected no significant interaction between wing length and treatment (*F*_(1,5)_ = 0.9, *P* = 0.49), indicating equivalent regression slopes for each treatment, while comparison of the adjusted treatment means indicated that clutch sizes significantly differed among treatments (*F*_(5,127)_ = 13.0, *P* < 0.0001). The total number of mature eggs produced by gnotobiotic females colonized by *Comamonas* did not significantly differ from the number of mature eggs produced by conventional females, whereas females colonized by *Aquitalea*, *Sphingobacterium, Chryseobacterium* and *Paenibacillus* all produced fewer mature eggs (Fig. 2b). Across all treatments, *A. atropalpus* females laid a majority of the mature eggs they produced (Fig. 2c). For both *A. aegypti* and *A. atropalpus,* hatch rates of eggs laid by gnotobiotic females exceeded 90 % and did not differ from eggs laid by conventionally reared females. Altogether then, treatment had no effect on egg production by *A. aegypti,* while in *A. atropalpus* the main difference between treatments was in the number of mature eggs females produced and laid rather than the viability of laid eggs.

**Fig. 3:**
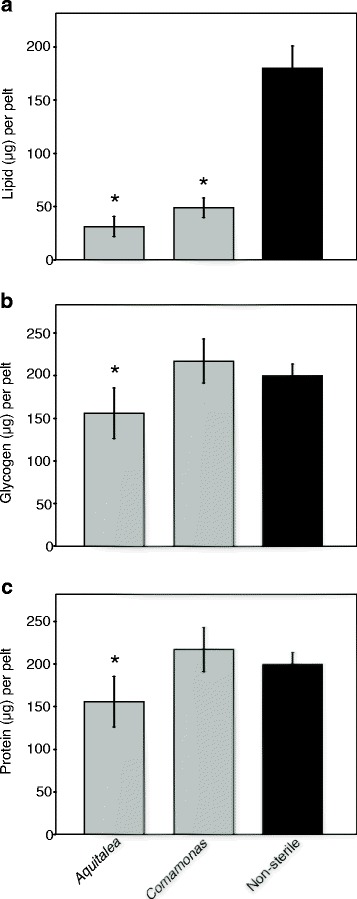
Total lipid (**a**), glycogen (**b**) and protein **c** in *Aedes atropalpus* adult females from larvae that were inoculated with *Aquitalea, Comamonas,* or conventionally reared (non-sterile). Lipid (ANOVA: *F*_(3,35)_ = 96.0, *P* < 0.0001), glycogen (ANOVA: *F*_(3,35)_ = 6.2, *P* = 0.002), and protein (ANOVA: *F*_(3,35)_ = 6.2, *P* = 0.002) significantly differed among treatments. For each nutrient, an asterisk above a bar indicates means significantly differ from the non-sterile control (Dunnett’s test; *P* < 0.01). Two adult females with gut removed were analyzed per replicate with 10 replicates analyzed for each treatment and nutrient. Bars indicate mean values with 95 % confidence intervals

Previous studies have measured the nutrient reserves (lipid, glycogen, protein) in conventionally reared *A. aegypti* and *A. atropalpus* females at adult emergence [10, 15]. We did not measure nutrient levels again in *A. aegypti* for this study because no differences in egg production were detected between gnotobiotic and conventionally reared females. However, we did compare nutrient reserves between gnotobiotic *A. atropalpus* colonized by *Aquitalea* or *Comamonas* and conventionally reared females. We also focused on these treatments because survival and adult female sizes were similar but egg production was lower in *Aquitalea-*colonized females. Results showed that stored lipid was higher in newly emerged conventional females than gnotobiotic females colonized by *Comamonas*, but protein and glycogen were equivalent (Fig. 3). In contrast, lipid, protein and glycogen levels were all higher in conventional than gnotobiotic females colonized by *Aquitalea* (Fig. 3).

### Bacterial loads in *A. aegypti* and *A. atropalpus* are similar

**Fig. 4:**
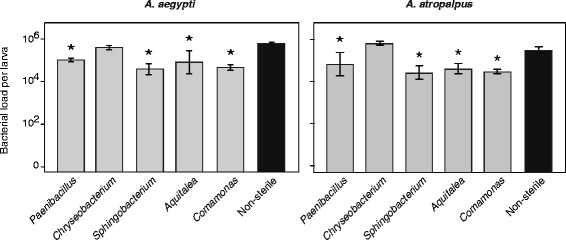
Bacterial loads in fourth instar gnotobiotic larvae colonized by a single bacterium and conventionally reared (non-sterile) fourth instars as measured by plate counts. Each gnotobiotic treatment and the conventionally reared (non-sterile) control are indicated on the X-axis. A minimum of 4 individuals was assayed per treatment. Bars indicate mean bacteria per larva with 95 % confidence intervals. Bacterial loads overall significantly differed among treatments for *Aedes aegypti* (ANOVA: *F*_(5,18)_ = 13.9, *P* < 0.0001) and *A. atropalpus* (ANOVA: *F*_(5,34)_ = 12.4, *P* < 0.0001). Asterisks (*) indicate treatments that significantly differ from conventionally reared larvae as determined by a post hoc Dunnett’s test (*P* < 0.01)

One possible explanation for the effects different gut community members have in gnotobiotic *A. aegypti* versus *A. atropalpus* is that bacterial abundance differed*.* We therefore assessed this by plating homogenates of larvae or adults after surface sterilization, which yielded CFUs per individual. Since larvae inoculated with *Microbacterium* or *Leucobacter* all died as first instars, we assayed first instars for these treatments at three days post-inoculation. Plating assays yielded no colonies from any larva, which in light of the data in Additional file 1: Figure S1 indicated that *A. aegypti* and *A. atropalpus* first instars ingested these species but they failed to colonize. Since inoculation with the other isolates resulted in most larvae molting but varying numbers of *A. atropalpus* developing into adults, we first compared bacterial load in larvae that had just molted to the final (fourth) instar (0–6 h). PCR assays as in Additional file 1: Figure S1 showed that gnotobiotic *A. aegypti* and *A. atropalpus* fourth instars both contained the bacterium they were inoculated with. Colony counts indicated that each gnotobiotic treatment except *Chryseobacterium* contained fewer viable bacteria per larva than the conventionally reared (non-sterile) positive control for both mosquito species (Fig. 4).

**Fig. 5:**
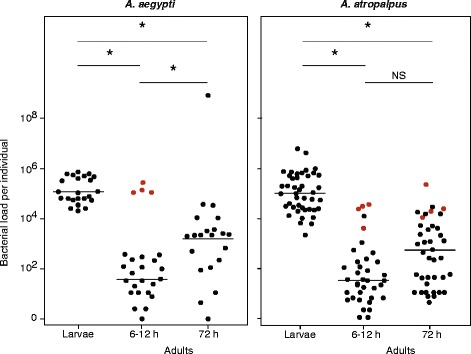
CFU counts in 24 h old fourth instars (Larvae), 6–12 h adult females, and 72 h adult females. Treatments and the number of individuals analyzed per life stage are the same as in Fig. 4. For *A. aegypti,* bacterial loads significantly differed among treatments in larvae (ANOVA: *F*_(5,18)_ = 13.9, *P* < 0.0001) and 6–12 h adults (ANOVA: *F*_(5,22)_ = 19.6, *P* < 0.0001), but did not differ in 72 h adults (ANOVA: *F*_(5,16)_ = 1.6, *P* = 0.21). Between stage comparisons indicated that bacterial loads significantly differed between larvae, 6–12 h adults and 72 h adults (ANOVA: *F*_(2,71)_ = 31.6, *P* < 0.0001; followed by a Tukey-Kramer HSD test). For *A. atropalpus,* bacterial loads significantly differed among treatments in larvae (ANOVA: *F*_(5,34)_ = 12.4, *P* < 0.0001), 6–12 h adults (ANOVA: *F*_(5,33)_ = 8.9, *P* < 0.0001) and 72 h adults (ANOVA: *F*_(5,36)_ = 6.8, *P* = 0.0002). Between stage comparisons indicated that larvae (*) had higher bacterial loads than 6–12 h or 72 h adults, which did not differ from one another (NS) (ANOVA: *F*_(1,118)_ = 86.6, *P* < 0.0005; followed by a Tukey-Kramer HSD test). For *A. aegypti* adults (6–12 h) and *A. atropalpus* adults (6–12 and 72 h)*,* Dunnett’s tests indicated treatment differences were due to higher colony counts for gnotobiotic individuals colonized by *Chryseobacterium* (*red data points*)

We then compared these larval data to bacterial loads in newly emerged (6–12 h) and 72 h old adults. For *A. aegypti,* 72 h old females had also blood-fed 24 h previously. Like the larval samples, each gnotobiotic treatment plus the non-sterile control was measured in at least four individuals for the two adult sample times. For 6–12 h old and 72 h old adults of both species, colony counts for each gnotobiotic treatment did not differ from the non-sterile control except for *Chryseobacterium* which was more abundant (Fig. 5). Merging the data from each treatment and comparing between stages for each species indicated that bacterial loads in *A. aegypti* were highest in fourth instars, lowest in 6–12 h adults and intermediate in 72 h adults (Fig. 5). In *A. atropalpus,* bacterial loads were also highest in fourth instars but no difference was detected between 6–12 h and 72 h adults (Fig. 5).

## Discussion

*Aedes aegypti* and *A. atropalpus* females are similar in size when reared as larvae under the same conditions, but *A. aegypti* emerges with lower nutrient reserves as measured by stored glycogen and protein [10, 15]. For both species, results in the literature also indicate the nutrients consumed by larvae affect adult size and the average number of eggs per clutch females lay [10, 30]. This finding suggests nutrient based thresholds play a role in egg production [10, 13, 15, 16]. In effect, *A. aegypti* females emerge with insufficient nutrient reserves and enter an arrested previtellogenic state that blood-feeding overcomes by providing additional nutrients. *Aedes atropalpus* females in contrast emerge with sufficient nutrients to initiate egg formation without blood-feeding. Enhanced nutrient acquisition along with genetic factors has also been implicated in anautogenous *versus* autogenous reproduction in other species [14, 31–35].

An earlier study used antibiotic treatment to determine whether gut bacteria in adult *A. aegypti* affect blood meal digestion [36]. Treatment with carbenicillin and tetracycline reduced the abundance of culturable bacteria in the midgut, which correlated with slower breakdown of the blood meal bolus and small, but statistically significant reductions in the number of eggs females laid. Identification of an *Enterobacter* and *Serratia* species with hemolytic activity further supported a role for the gut microbiota in blood meal processing. Outside of this work, however, what role if any the gut microbiota plays in female-associated fitness including egg production is unknown in mosquitoes [37], which is why we conducted this study.

Our previous results showed that certain genera of Actinobacteria (*Microbacterium* and *Leucobacter*), Bacteroidetes (*Chryseobacterium* and *Sphingobacterium*), Betaproteobacteria (*Comamonas* and *Aquitalea*) and Firmicutes (*Paenibacillus*) are present in the digestive tracts of both *A. aegypti* and *A. atropalpus* larvae when reared under identical conditions in the laboratory [21]. The relative abundance of these community members, however, differs with Bacteroidetes and Actinobacteria predominating in *A. aegypti* and Betaproteobacteria predominating in *A. atropalpus.* These differences guided our choice of the gut bacteria used in this study to compare the effects individual members have on fitness relative to conventionally reared individuals with a mixed community of bacteria or axenic larvae with no gut bacteria.

The first part of our study corroborates prior findings by showing that axenic *A. aegypti* and *A. atropalpus* larvae die as first instars. They also show that *Microbacterium* and *Leucobacter* fail to colonize larvae of either species, which likewise results in no survival. This finding strongly suggests *Microbacterium* and *Leucobacter* present in our conventionally reared larvae require other bacteria to survive in *A. aegypti* and *A. atropalpus.* The other community members we tested resulted in nearly all *A. aegypti* larvae developing into adults that did not differ from adults of conventionally reared larvae in development time, size or the number of mature eggs females produced after consuming a blood meal. In contrast, only the two Betaproteobacteria (*Aquitalea* and *Comamonas*) tested in *A. atropalpus* supported survival, development times and adult body sizes that were similar to those of conventionally reared larvae. Only *A. atropalpus* larvae colonized by *Comamonas* produced the same number of eggs per first clutch as conventionally reared females. The three other community members we tested (*Paenibacillus*, *Chryseobacterium* and *Sphingobacterium*) are more abundant in conventionally reared *A. aegypti* than *A. atropalpus*. Each of these *Paenibacillus*, *Chryseobacterium* and *Sphingobacterium* species colonized *A. atropalpus* to produce gnotobiotic larvae and increased survival relative to axenic larvae. However, survival and other fitness measures were also much lower than those of conventionally reared *A. atropalpus*.

Taken together, these results indicate that several members of the gut community in *A. aegypti* larvae support development and egg production to levels that are comparable to conventionally reared individuals with a mixed bacterial community. They also suggest the differential abundance of these bacteria in conventionally reared *A. aegypti* larvae does not reflect the ability of these community members to rescue larval development or egg production by adult females after a blood meal. This is not the case though for *A. atropalpus,* where the two Betaproteobacteria we tested are more abundant in conventionally reared larvae and also had a more positive effect on survival, size and egg production in gnotobiotic larvae. Thus, one important element of autogeny in *A. atropalpus* could be greater dependence on the composition of the gut microbiota for development and reproduction. In addition, the impacts of gut bacteria on nutrient reserves in *A. aegypti* are potentially compensated for by the extra nutrients females obtain from a blood meal.

Gnotobiotic *A. atropalpus* colonized by *Comamonas* emerged with similar stores of glycogen and protein but significantly lower total lipid than conventionally reared females. In contrast, gnotobiotic females colonized by *Aquitalea* emerged with lower glycogen, protein, and lipid stores. Reduced nutrient uptake and metabolism in axenic or gnotobiotic backgrounds have been reported for mice, zebrafish, *Daphnia* and *Drosophila* [38–42]. The gut microbiota of *Drosophila* is also known to promote growth by modulating nutrient sensing through the target of rapamycin and insulin signaling pathways, which intersect to regulate metabolism [43, 44]. Thus, the higher lipid stores in conventionally reared females suggest other community members besides *Aquitalea* or *Comamonas* are required for normal lipid storage. However, lower lipid levels in *Comamonas-*colonized females did not correlate with reduced first clutch sizes, which suggests stored protein is the more important determinant for production of a first clutch. The importance of stored protein may also underlie why gnotobiotic females colonized by *Aquitalea* produced smaller clutches.

Most insects including mosquitoes lay eggs that by dry weight consist primarily of protein but also 30–40 % lipid that is predominantly triacylglycerol (TAG) [9, 45]. TAG is synthesized from free fatty acids and glycerol but insect eggs exhibit little or no de novo fatty acid synthetic activity. Thus, yolk protein and TAG are both imported from the fat body into oocytes during oogenesis [45]. In *A. aegypti*, digestion of a blood meal primarily provides amino acids, which suggests TAG packaged into eggs during the first gonadotropic cycle derives primarily from stores in the fat body while yolk protein is produced from a combination of nutrient stores and blood meal derived amino acids [9]. In the case of *A. atropalpus,* however, TAG and yolk protein must both come from teneral reserves in the fat body. The mobilization of nutrient stores in *A. atropalpus* colonized by *Comamonas* is of interest because these females have lower stored lipid relative to females of conventionally reared larvae, but the number of mature eggs produced by both sets of females was similar, as were their protein and carbohydrate stores. How these gnotobiotic females accomplish this is unclear. One possibility is their lower lipid reserves are sufficient to mobilize as TAG and produce a comparable number of mature eggs as conventionally reared females. Alternatively, excess carbohydrate or protein stores may be catabolized to acetyl-CoA, which is the precursor for fatty acid synthesis and thus TAG importation, and would allow females to produce a similar number of mature eggs as conventionally reared females with larger lipid reserves.

Excluding *Microbacterium* and *Leucobacter*, our measures of bacterial abundance in gnotobiotic *A. aegypti* and *A. atropalpus* showed that the average number of living bacteria per individual was either similar or slightly lower in most gnotobiotic treatments when compared to conventionally reared controls. This finding indicates each of the community members we tested proliferated in the absence of other community members, but usually did not exceed the total abundance of bacteria in conventional larvae that host a mixed bacterial community. These results indicate that the higher survival and egg production of gnotobiotic *A. atropalpus* colonized by *Comamonas* was not due to this bacterium being more abundant in the guts of larvae or adults than the other community members we tested. Thus, other features of this bacterium likely underlie its positive effects for *A. atropalpus*. These data also indicate that bacterial abundance in gnotobiotic and conventionally reared individuals is lower in adults than fourth instar larvae, which is a feature previously noted for *A. aegypti* [21].

## Conclusions

Results of this study show that *A. aegypti* and *A. atropalpus* larvae both require gut bacteria to develop into adults, but individual gut bacteria differentially affect development and reproduction by both species when reared as gnotobiotic larvae. In particular, autogenous egg production by *A. atropalpus* has a greater dependence on the specific members of the gut microbiota, while the added nutrients females obtain from blood-feeding potentially make *A. aegypti* females less dependent on the composition of the gut microbiota.

## Abbreviations

CFU, Colony forming unit; ILPs, Insulin-like peptides; OEH, Ovary ecdysteroidogenic hormone

## Additional file

Additional file 1: Figure S1. Axenic *A. aegypti* and *A. atropalpus* first instars rapidly ingested bacteria. Conventional first instars were hatched in open containers containing distilled water and sterilized diet (Non-sterile). Axenic first instars were hatched in fully sterile conditions and fed sterilized diet (Axenic). Other axenic larvae were fed sterilized diet plus the indicated bacterial isolate. For each treatment, DNA was isolated from a pooled sample of 10 larvae that was collected 6 h post-inoculation after repeated washing and surface sterilization per Coon *et al.* [21]. DNA was also isolated from cultures of each bacterial isolate. DNA samples were then used as templates with universal or genus-specific primers (see Methods and Table S2). The agarose gel shows ethidium bromide stained PCR products. Lane 1, molecular mass markers labeled in base pairs (bp); Lane 2, universal primers plus DNA from conventional larvae; Lane 3, universal primers plus DNA from axenic larvae; Lane 3-4, *Chryseobacterium-*specific primers plus template from *Chryseobacterium* (Control) or axenic first instars inoculated with *Chryseobacterium* (Larva). The same treatments are then shown for *Sphingobacterium* (Lanes 5-6)*, Microbacterium* (Lanes 7-8)*, Leucobacter* (Lanes 9-10)*, Paenibacillus* (Lanes 11-12)*, Aquitalea* (Lanes 13-14)*,* and *Comamonas* (Lanes 15-16). This experiment was repeated four times with independently collected samples and each time yielded identical outcomes for both *A. aegypti* and *A. atropalpus.* **Table S1.** Bacterial isolates used in the study. **Table S2.** Primers designed and used during the study. (PDF 282 kb)
