# Supplementary material for: Gut bacteria differentially affect egg production in the anautogenous mosquito Aedes aegypti and facultatively autogenous mosquito Aedes atropalpus (Diptera: Culicidae)
Source: Parasit Vectors. 2016 Jun 30;9:375. doi: 10.1186/s13071-016-1660-9 (PMC4929711; doi:10.1186/s13071-016-1660-9)
Supplement: Additional file 1: Figure S1. — Axenic A. aegypti and A. atropalpus first instars rapidly ingested bacteria. Conventional first instars were hatched in open containers containing distilled water and sterilized diet (Non-sterile). Axenic first instars were hatched in fully sterile conditions and fed sterilized diet (Axenic). Other axenic larvae were fed sterilized diet plus the indicated bacterial isolate. For each treatment, DNA was isolated from a pooled sample of 10 larvae that was collected 6 h post-inoculation after repeated washing and surface sterilization per Coon et al. [21]. DNA was also isolated from cultures of each bacterial isolate. DNA samples were then used as templates with universal or genus-specific primers (see Methods and Table S2). The agarose gel shows ethidium bromide stained PCR products. Lane 1, molecular mass markers labeled in base pairs (bp); Lane 2, universal primers plus DNA from conventional larvae; Lane 3, universal primers plus DNA from axenic larvae; Lane 3-4, Chryseobacterium-specific primers plus template from Chryseobacterium (Control) or axenic first instars inoculated with Chryseobacterium (Larva). The same treatments are then shown for Sphingobacterium (Lanes 5-6), Microbacterium (Lanes 7-8), Leucobacter (Lanes 9-10), Paenibacillus (Lanes 11-12), Aquitalea (Lanes 13-14), and Comamonas (Lanes 15-16). This experiment was repeated four times with independently collected samples and each time yielded identical outcomes for both A. aegypti and A. atropalpus. Table S1. Bacterial isolates used in the study. Table S2. Primers designed and used during the study. (PDF 282 kb) [file 13071_2016_1660_MOESM1_ESM.pdf]

Supporting Information

Fig. S1. Axenic *A. aegypti* and *A. atropalpus* first instars rapidly ingested bacteria. Conventional first instars were hatched in open containers containing distilled water and sterilized diet (Non-sterile). Axenic first instars were hatched in fully sterile conditions and fed sterilized diet (Axenic). Other axenic larvae were fed sterilized diet plus the indicated bacterial isolate. For each treatment, DNA was isolated from a pooled sample of 10 larvae that was collected 6 h post-inoculation after repeated washing and surface sterilization per Coon *et al.* [21]. DNA was also isolated from cultures of each bacterial isolate. DNA samples were then used as templates with universal or genus-specific primers (see Methods and Table S2). The agarose gel shows ethidium bromide stained PCR products. Lane 1, molecular mass markers labeled in base pairs (bp); Lane 2, universal primers plus DNA from conventional larvae; Lane 3, universal primers plus DNA from axenic larvae; Lane 3-4, *Chryseobacterium*-specific primers plus template from *Chryseobacterium* (Control) or axenic first instars inoculated with *Chryseobacterium* (Larva). The same treatments are then shown for *Sphingobacterium* (Lanes 5-6), *Microbacterium* (Lanes 7-8), *Leucobacter* (Lanes 9-10), *Paenibacillus* (Lanes 11-12), *Aquitalea* (Lanes 13-14), and *Comamonas* (Lanes 15-16). This experiment was repeated four times with independently collected samples and each time yielded identical outcomes for both *A. aegypti* and *A. atropalpus.*

Figure S1.

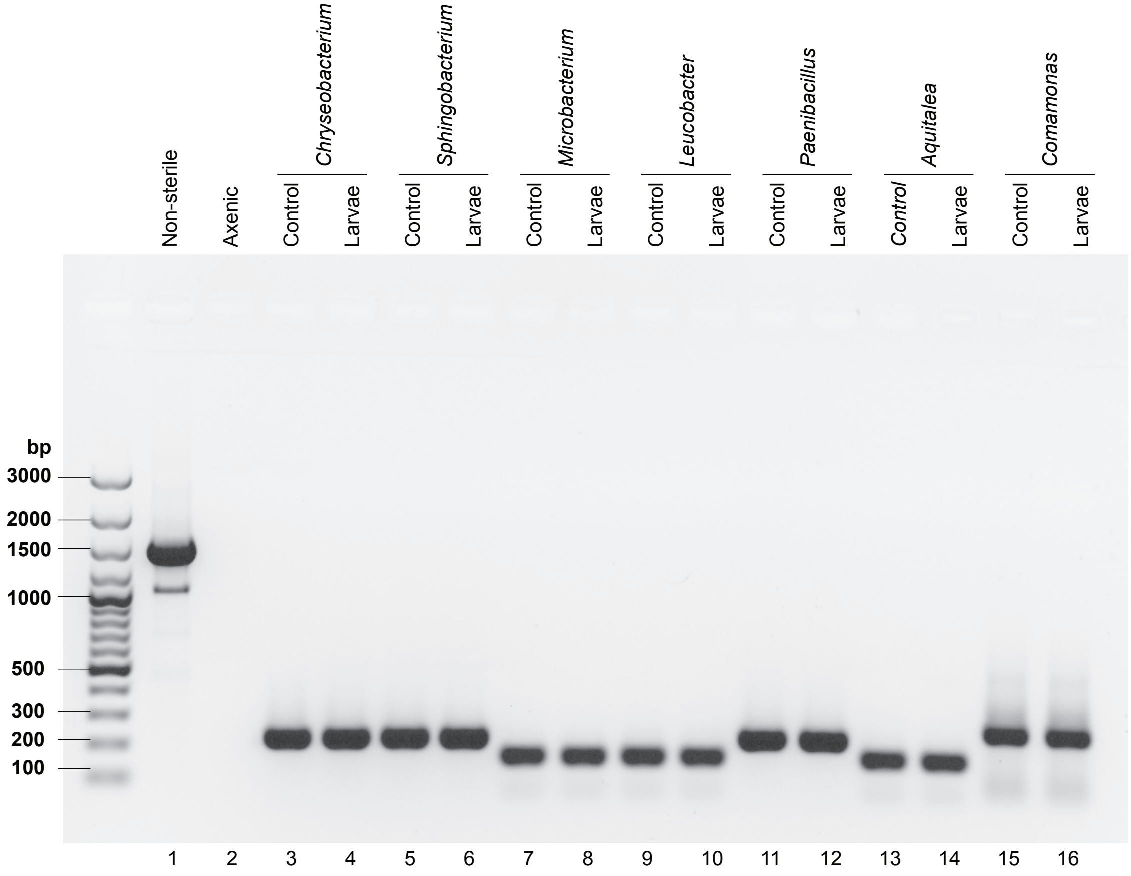
Non-sterile
Axenic
Chryseobacterium
Sphingobacterium
Microbacterium
Leucobacter
Paenibacillus
Aquitalea
Comamonas
Control
Larvae
bp
3000
2000
1500
1000
500
300
200
100
1 2 3 4 5 6 7 8 9 10 11 12 13 14 15 16

Table S1. Bacterial isolates used in the study.

**Mosquito species isolate was collected from**	**Colony color and morphology**	**Culture medium**	**Genus**
A. aegypti	White/pink + sec	Nutrient agar (BHI)	*Paenibacillus*
A. aegypti	Yellow	Nutrient agar (BHI)	*Chryseobacterium*
A. aegypti	White	Nutrient agar (BHI)	*Sphingobacterium*
A. aegypti	White (small)	Blood agar (TSA base)	*Microbacterium*
A. aegypti	White (small)	Nutrient agar (BHI)	*Leucobacter*
A. atropalpus	Green	R2A	*Aquitalea*
A. atropalpus	White (small) + sec	R2A	*Comamonas*

Table S2. Primers designed and used during the study.

Name	Sequence	Target gene	Target	Tm (°C)	Reference
General primers (product > 300 bp)					
27F	GAGAGTTTGATCCTGGCTCAG	16S rRNA	Most bacteria	50	1
1492R	GGTTACCTTGTTACGACTT	16S rRNA	Most bacteria	50	1
1492F	AAGTCGTAACAAGGTAACC	16S rRNA	Most bacteria	55	1
129R	GGTTBCCCCATTCRG	23S rRNA	Most bacteria	55	2
Quantitative PCR primers (product < 300 bp)					
*HDA1	ACTCCTACGGGAGGCAGCAGT	16S rRNA	Most bacteria	55	3
*HDA2	GTATTACCGCGGCTGCTGGCA	16S rRNA	Most bacteria	55	3
Aqui-qF	GTTGAGCACTCTAATGGGAC	16S-23S ITS	*Aquitalea* sp.	55	
Aqui-qR	TCGGTTTTATGAGATTGGCT	16S-23S ITS	*Aquitalea* sp.	55	
Chryseo-qF	GAAACTGCCATTGATACTGC	16S-23S ITS	*Chryseobacterium* sp.	55	
Chryseo-qR	CCCAAAAACGAGTTAGCATC	16S-23S ITS	*Chryseobacterium* sp.	55	
Coma-qF	GAGTCGAAAATCAGCGTTGC	16S-23S ITS	*Comamonas* sp.	55	
Coma-qR	GCACAAGAACCCAAGCAACT	16S-23S ITS	*Comamonas* sp.	55	
Micro-qF	AACTAGTTGTGGGGTCCA	16S-23S ITS	*Microbacterium* sp.	55	
Micro-qR	TGTATGTCAAGCCTTGGTAA	16S-23S ITS	*Microbacterium* sp.	55	
Paeni-qF	TAGGTGTTGGGGATTCGAT	16S-23S ITS	*Paenibacillus* sp.	55	
Paeni-qR	CACCTGTCTCCTCTGTCC	16S-23S ITS	*Paenibacillus* sp.	55	

*Universal primers were used for qPCR analysis of *Sphingobacterium*- and *Leucobacter*-associated mosquitoes

1. Lane DJ. 16S/23S rRNA sequencing. In: Stackebrandt E, Goodfellow M, editors. Nucleic acid techniques in bacterial systematics. New York:John Wiley and Sons; 1991. p. 115-175.

2. Gurtler V, Stanisich VA. New approaches to typing and identification of bacteria using the 16S-23S rDNA spacer region. Microbiology. 1996;142:3-16.

3. Walter J, Tannock GW, Tilsala-Timisjarvi A, Rodtong S, Loach DM, Munro K, Alatossava T. Detection and identification of gastrointestinal *Lactobacillus* species by using denaturing gradient gel electrophoresis and species-specific PCR primers. Appl Environ Microbiol. 2000;66:297-303.
